# Sex Differences in a Rodent Model of HIV-1-Associated Neuropathic Pain

**DOI:** 10.3390/ijms20051196

**Published:** 2019-03-09

**Authors:** Josée Guindon, Henry Blanton, Seth Brauman, Kelsey Donckels, Madhusudhanan Narasimhan, Khalid Benamar

**Affiliations:** Department of Pharmacology and Neuroscience, Texas Tech University Health Sciences Center, School of Medicine Lubbock, Lubbock, TX 79430, USA; josee.guindon@ttuhsc.edu (J.G.); henry.blanton@ttuhsc.edu (H.B.); braumansethc@sau.edu (S.B.); kelsey.donckels@ttu.edu (K.D.); madhu.narasimhan@ttuhsc.edu (M.N.)

**Keywords:** neuropathic pain, gp120, ovarian hormones, estrous-cycle, ovariectomized, HIV

## Abstract

Worldwide, women account for approximately 51% of human immunodeficiency virus-1 (HIV) seropositive individuals. The prevalence of neuropathic pain among individuals with HIV and a lack of preclinical data characterizing sex differences prompted us to address this knowledge gap. C57BL/6 male and female mice received multiple intrathecal injections of HIV-glycoprotein 120 (gp120), followed by determination of mechanical allodynia and thermal hypersensitivity for four weeks. The influence of ovarian hormones in the gp120 pain model was evaluated by comparison of ovariectomized (OVX) mice versus sham control. We found that gp120-induced neuropathic pain-like behaviors are sex-dependent. Female mice showed both increased mechanical allodynia and increased cold sensitivity relative to their male counterparts. The OVX mice showed reduced pain sensitivity compared to sham, suggesting a role of the ovarian hormones in sex differences in pain sensitivity to gp120. Gp120-induced neuropathic pain caused a shift in estrous cycle toward the estrus phase. However, there is a lack of clear correlation between the estrous cycle and the development of neuropathic pain-like behaviors during the four week recording period. This data provided the first evidence for sex differences in a rodent model of HIV-related neuropathic pain, along with a potential role of ovarian hormones.

## 1. Introduction

Human immunodeficiency virus-1 (HIV)-associated neuropathic pain is a debilitating condition affecting 55%–67% of the 36.9 million people living with HIV worldwide (available online: http://www.who.int/hiv/data/en/). HIV-associated neuropathic pain can occur at any time during the entire process of the disease; it remains a common and debilitating condition frequently reported in the clinics, despite the availability of combination antiretroviral therapy (cART) [1]. However, in a vast majority of the cases with HIV infection, neuropathic pain and cognitive impairment persist despite the use of cART [1], indicating a need to better understand the mechanistic basis that extends beyond the direct effects of the virus. Indeed, a new spectrum of problems is emerging as a result of the action of HIV-viral proteins. Foremost among these viral proteins, there is the envelope glycoprotein gp120 (gp120). HIV enters target cells by binding its gp120 to the CD4 receptor and its co-receptors, such as C–C chemokine receptor type 5 (CCR5) or C–X–C chemokine receptor type 4 (CXCR4). Gp120 has been found in the brains of patients with HIV encephalitis who also suffer from dementia [2]. In the spinal cord dorsal horn, gp120 has been found at greater concentrations among pain-positive versus pain-negative HIV patients [3]. The involvement of this viral protein in the pathogenesis of HIV-related neuropathic pain has been demonstrated in animal models [3,4,5].

Clinically, women make up the large majority of chronic pain patients. In fact, there is now consensus from laboratory experiments to suggest that when differences are seen, women are more sensitive to pain than men [6]. In addition, a positive association between women and the presence of pain in HIV has been reported [7,8,9]. While some studies reported an absence of correlation [10,11], these divergent findings may be due to variations in sample composition and/or pain assessment, including the use of non-standardized measures and small sample sizes.

The present study was designed to investigate the effects of sex variable on the development, duration, and severity of neuropathic pain-like behaviors induced by gp120, and any possible role played by ovarian hormones. This research will lay the groundwork for future mechanistic analyses of neurobiological sex differences within the context of HIV-related neuropathic pain.

## 2. Results

### 2.1. Sex Difference in gp120-Induced Mechanical Allodynia and Cold Hypersensitivity

**Figure 1:**
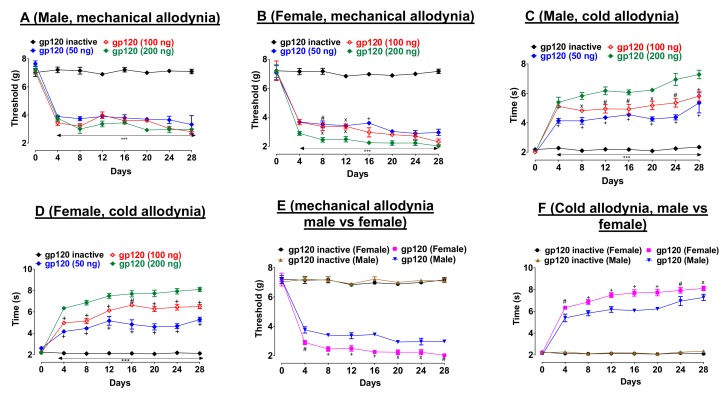
Development of mechanical allodynia and cold hypersensitivity to gp120 in male and female mice. Hind paw withdrawal thresholds to an electronic von Frey device and an acetone drop in male (**A**,**C**) and female (**B**,**D**) following intrathecal (i.t.) gp120 injection or heat-inactivated gp120 (*n* = six). Compared to male mice, female mice showed lower pain thresholds in von Frey (**E**) and acetone (**F**) tests. Statistical significance of differences between gp120 and gp120 inactive or between male (gp120, 200 ng) and female (gp120, 200 ng) were conducted using two-way ANOVA, multiple comparisons, Bonferroni post hoc. *** *p* < 0.0001 vs. gp120 inactive (**A**–**D**) group. + *p* < 0.001, # *p* < 0.01, x *p* < 0.05 comparing gp120 at 200 ng compared to 100 and 50 ng (**A**–**D**) or comparing gp120 at 200 ng between male to female (**E**,**F**). Data are expressed as mean ± S.E.M. (*n* = six per group).

First, we determined the dose-response effects of gp120 on pain thresholds. Three injections of gp120 (50, 100, or 200 ng/10 µL) on days one, three, and seven decreased mechanical allodynia thresholds (von Frey test) relative to heat-inactivated gp120 throughout the duration of this experiment, in both male (Figure 1A, F_21,160_ = 11.4, *p* < 0.001) and female mice (Figure 1B, F_21,160_ = 10.82, *p* < 0.001). Compared to 50 and 100 ng doses, gp120 at 200 ng induced the highest effect in male and female mice (Figure 1A (F_14,120_ = 0.8, *p* < 0.001) and B (F_21,120_ = 0.91, *p* < 0.001), respectively).

Similarly, gp120 (50, 100, or 200 ng/10 µL) induced cold hypersensitivity (acetone test) relative to heat-inactivated gp120 throughout the duration of this experiment in male (Figure 1C, F_21,160_ = 9.002, *p* < 0.001) and female (Figure 1D, F_21, 160_ = 18.39, *p* < 0.001) mice. Compared to 50 and 100 ng doses, gp120 at 200 ng induced the highest effect in male and female mice (Figure 1C (F_14,120_ = 2.29, *p* < 0.001), and D (F_14,120_ = 2.29, *p* < 0.001)). Second, we compared gp120-induced neuropathic pain-like behaviors between male and female. In both tests, we observed a noticeable sex-difference. In particular, female mice showed lower pain thresholds compared to their male counterparts in both von Frey (Figure 1E, F_21,190_ = 24.67, *p* < 0.001) and acetone tests (Figure 1F, F_21,160_ = 32.88, *p* < 0.001) during the four-week testing period.

### 2.2. Lower Pain Sensitivity in ovariectomized (OVX) Mice Relative to Sham Mice Following gp120 Induction of Neuropathic Pain-Like Behaviors

**Figure 2:**
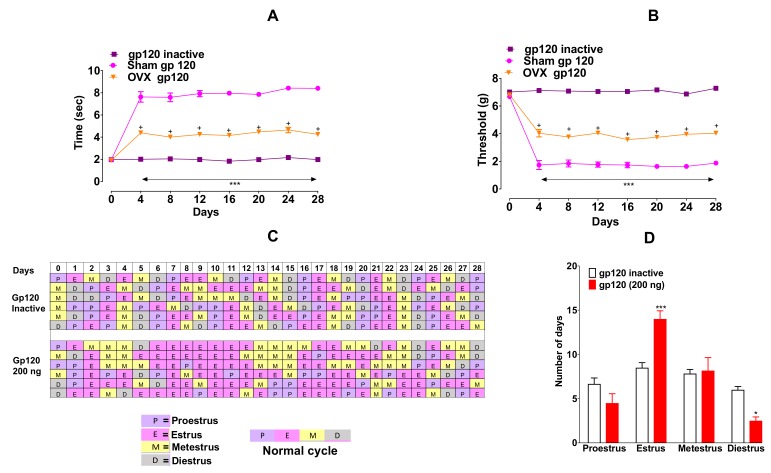
Ovariectomized (OVX) mice show less pain sensitivity compared to sham. Gp120 (200 ng/10 µL, (i.t) induced cold hypersensitivity (2A) and mechanical allodynia and (2B) in both sham and OVX mice. OVX mice showed lower pain sensitivity in mechanical allodynia (**A**) and thermal hypersensitivity (**B**) compared to sham. *** *p* < 0.0001, + *p* < 0.05 (two-way ANOVA, multiple comparisons, Bonferroni post hoc). Data are expressed as mean ± S.E.M. (*n* = six per group). Gp120 altered the estrous cycle (**C**). Time spent in estrus was prolonged (**D**, *p* < 0.001) and diestrus was reduced (**D**, *p* < 0.01) compared to heat-inactivated gp120. *** *p* < 0.001, * *p* < 0.05 vs. gp120 inactive group (two-way ANOVA, multiple comparisons, Bonferroni post hoc). Data are expressed as mean ± S.E.M. (*n* = 6 per group).

To determine any role of ovarian hormones in sex differences in pain sensitivity to gp120 (200 ng/10 µL, i.t), we evaluated the effect of this viral protein in OVX and sham mice for four weeks. OVX mice showed lower pain sensitivity in mechanical allodynia (Figure 2A, F_14,48_ = 39.36, *p* < 0.001) and thermal hypersensitivity (Figure 2B, F_14,48_ = 35.59, *p* < 0.001) compared to sham, *** *p* < 0.0001 vs. sham.

We also evaluated the estrous cyclicity by counting the number of days spent in each stage. We found that the administration of gp120 ceased the normal estrous-cycle (Figure 1C). In particular, gp120 markedly prolonged the time spent in estrus stage (building-up phase) (Figure 2D, F_3,40_ = 11.57, *p* < 0.001) with a significantly reduced diestrus (Figure 2D, F_3,40_ = 11.57, *p* < 0.01) duration compared to heat-inactivated gp120. However, we did not observe a correlation between the shift in the cycle and development of neuropathic pain-like behaviors.

We also evaluated the estrous cyclicity by counting the number of days spent in each stage. We found that the administration of gp120 interfered the normal estrous-cycle (Figure 1C). In particular, gp120 markedly prolonged the time spent in estrus stage (building-up phase) (Figure 2D, *p* < 0.001) with a significantly reduced diestrus (Figure 2D, *p* < 0.01) duration compared to heat-inactivated gp120. However, we did not observe a correlation between the shift in the cycle and development of neuropathic pain-like behaviors.

Gp120 altered the estrous cycle (Figure 2C). Time spent in estrus was prolonged (Figure 2D, *p* < 0.001) and diestrus was reduced (Figure 2D, *p* < 0.01) compared to heat-inactivated gp120. *** *p* < 0.001, * *p* < 0.05 vs. gp120 inactive group (two-way ANOVA, multiple comparisons, Bonferroni post hoc). Data are expressed as mean ± SEM (*n* = 6 per group).

## 3. Discussion

In light of our research, we are reporting three key findings. First, gp120-induced neuropathic mechanical and cold allodynia is sex-dependent. Females demonstrated lower pain thresholds compared to males. Second, ovarian hormones seem to play a role in sex differences to gp120-induced neuropathic pain-like behaviors. Third, gp120 induced a shift in the estrous-cycle toward the estrus state. However, there was an absence of a clear correlation between the estrous cycle and the development of neuropathic pain, when compared to behaviors during the four-week recording period.

We compared gp120-induced-neuropathic pain-like behaviors in male and female mice. We found that pain hypersensitivity to mechanical and cold stimuli was more pronounced in females compared to males during the four-week recording period. Our data demonstrate that the effect of this viral protein on pain sensitivity is sex-dependent; in addition, this biological variable plays a key role in the gp120 sensory pain action. Further, the removal of ovaries via OVX reduced pain hypersensitivity to the sham, which indicates a potential role for ovarian hormones on sex differences in pain hypersensitivity to gp120. While the action of gp120 on ovarian hormones that exerts a differential influence on the main endogenous system involved in pain control (such as opioid and cannabinoid) may possibly have a significant impact in the observed sex difference-based pain sensitivity, other mechanisms can also play a role. Previous studies show that gp120 induced overexpression of spinal astrocytes microglia immunoreactivity [4,5]. Microglia and immune cell (peripheral) play a role in chronic neuropathic pain conditions [12]. Recent evidence suggests that neuroimmune modulation of pain, an outcome of immune–neuron interaction may be divergent in males and females [13]. A recent study showed that female mice use T-cell-to-neuron signaling to cause pain hypersensitivity, while males use microglia-to-neuron signaling [14].

In the present study, we also observed that gp120 administration destabilized the estrous cycle. An elongated duration in estrus together with a shorter diestrus phase was consistently noted in gp120-administered animals. However, there is a lack of clear correlation between the estrous cycle and the development of neuropathic pain-like behaviors during the four-week recording period. The mechanisms driving altered cyclicity by gp120 is currently unknown. Gp120 may impair and/or abnormally interact with the neural control of the hypothalamic–pituitary–gonadal circuitry, leading to irregular estrous cycles, and affect the periodic reproductive cycling.

## 4. Materials and Methods

### 4.1. Animals

Female and male (8–10 weeks old), wild-type C57BL/6J mice obtained from Jackson Laboratories (Bar Harbor, ME, USA) were used. They were housed in groups of 3 for at least 1 week in an animal room maintained at 22 ± 1 °C and approximately 50% ± 5% relative humidity. Lighting was on a 12/12-h light/dark cycle (lights on at 7:00 and off at 19:00). The animals were allowed free access to food and water. All animal care and experimental procedures were (#14037, 2 August 2019) approved by the Institutional Animal Care and Use Committee of the Texas Tech University Health Sciences Center and conformed to the Guidelines of the National Institutes of Health on the Care and Use of Animals. TTUHSC’s animal facility is AAALAC approved.

### 4.2. Drugs and Preparation

Gp120_ADA_ (M-tropic strain) was obtained from Advanced Biotechnologies Inc. (Columbia, MD, USA). Gp120 was prepared in 0.1% rat serum albumin in sterile saline.

### 4.3. Behavioral Testing

Gp120 was administered intrathecally (i.t., lumbar puncture [15] at different doses of gp120 (50–200 ng/10 µL) in mice on days 1, 3, and 7. For the control group, we used inactive HIV gp120 (boiled).

### 4.4. Assessment of Mechanical Allodynia

Mechanical withdrawal thresholds were assessed using a digital Electrovonfrey (IITC Life Sciences, Woodland Hills, CA, USA) as previously reported [16]. Briefly stated, mice were placed in individual plastic cages on an elevated wire mesh platform, and were allowed to habituate to the testing apparatus for at least 15 min, or until exploratory behavior was no longer observed.

Force was applied to the mid-plantar region of the left and right hind paws. Two thresholds were taken for each paw. Force was applied to the mid-plantar region of the left and right hind paws. Two thresholds were measured for each paw. The force at which this response occurs is recorded automatically by the apparatus, and was designated as the “paw withdrawal threshold”.

### 4.5. Assessment of Cold Allodynia

Cold allodynia was assessed by applying a drop of room temperature acetone to the plantar surface of the hind paw as previously described [16]. After the animals stopped the exploratory behavior, one drop of acetone (approximately 20 μL) was applied through the mesh platform onto the plantar surface of the hind paw. Time of paw withdrawal responses were recorded.

### 4.6. Vaginal Lavage

Each mouse’s posterior was exposed until the vaginal area was visible. A 100–1000 µL pipette was used and 100 µL of distilled water was placed at the entrance of the vaginal canal. The liquid was aspirated into the canal and placed on a glass slide to allow the smear to completely dry at room temperature. Once dried, these estrous smears were stained immediately with crystal violet.

### 4.7. Ovariectomy

Mice were anesthetized with 4% isoflurane for induction and 2% for maintenance. A small longitudinal incision (15 mm) of the abdominal wall was performed followed by the incision of the fascia, which led to the exposition of internal organs [17]. Laparoscopy of the abdomen was performed to identify the ovaries. The horn was ligatured with absorbable silk (5.0) sutures followed by the incision of the horn above the suture. The fascia and the abdominal muscles were closed with a suture using absorbable silk (4.0), the skin was closed together using surgical staples.

### 4.8. Statistical Analysis

The data was expressed both as a mean and a standard error of the mean (SEM). Statistical analysis of the difference between groups for behavioral experiments was assessed using a two-way analysis of variance (ANOVA, prism 8), followed by Bonferroni multiple comparison test. *p* < 0.05 was taken as the significant level of difference.

## 5. Conclusions

In the current study, we provided new evidence concerning the role of sex differences in a rodent model of HIV-related neuropathic pain, along with the role of ovarian hormones in this process. Further studies would open up new avenues for mechanistic analyses of the underlying neurobiology sex difference. While we used gp120 from M-tropic stain to determine the sex differences in pain sensitivity, the role of others strains (e.g., T-tropic) still needs to be determined. The focus on gp120 does not negate the potential contribution of other viral proteins in the pathogenesis of neuropathic pain in the context of HIV.
